# Improving radiation physics, tumor visualisation, and treatment quantification in radiotherapy with spectral or dual‐energy CT

**DOI:** 10.1002/acm2.13468

**Published:** 2021-11-07

**Authors:** Matthijs Ferdinand Kruis

**Affiliations:** ^1^ Clinical Science CT Philips Healthcare Best The Netherlands

**Keywords:** conventional CT, dose calculation, dual‐energy CT, material decomposition, proton therapy, quantification, radiotherapy, tumor visibility, spectral CT

## Abstract

Over the past decade, spectral or dual‐energy CT has gained relevancy, especially in oncological radiology. Nonetheless, its use in the radiotherapy (RT) clinic remains limited. This review article aims to give an overview of the current state of spectral CT and to explore opportunities for applications in RT.

In this article, three groups of benefits of spectral CT over conventional CT in RT are recognized. Firstly, spectral CT provides more information of physical properties of the body, which can improve dose calculation. Furthermore, it improves the visibility of tumors, for a wide variety of malignancies as well as organs‐at‐risk OARs, which could reduce treatment uncertainty. And finally, spectral CT provides quantitative physiological information, which can be used to personalize and quantify treatment.

## 1 INTRODUCTION

Computed tomography (CT) has been around for over four decades and is indispensable in radiology. In 1983, the use of CT for radiotherapy (RT) treatment planning was first proposed,^1^ and since then CT gained a pivotal role within RT treatment simulation. Most contemporary RT departments have a dedicated CT system used as a RT simulator. The anatomical information from CT supports delineation of the treatment target and organs‐at‐risk (OAR), while the attenuation information serves dose calculation purposes.

CT techniques have developed significantly over the years, with spectral CT imaging being one of the most prominent recent innovations. Conventional CT reports the averaged attenuation of a polychromatic radiation beam in the patient, which makes the measurements dependent on tube output and body size due to beam hardening.^2^ However, since body attenuation of kilovoltage (kV) radiation is caused for over 85% by Compton scatter (CS) and the photoelectric effect (PE),^3^ a much more quantitative dataset can be created through dual‐energy (DE) CT.

By acquiring CT data at two different energy levels and decomposing these into two base images that can be used to describe the X‐ray interaction (such as CS/PE or effective atomic numbers [Z_eff_]/electron density [ED]), a quantitative dataset can be composed.^4^ These datasets can be recomposed into other quantitative maps like virtual monochromatic images (VMI) representing mono‐energetic photon energies at different kiloelectron Volt (keV) levels or single material decompositions,^5^

Initial decomposition can be done either in the image or in the sinogram domain. Decomposition in the image domain is the simplest solution, but since the reconstructions of the original input conceive conventional data, beam hardening artefacts are introduced during reconstruction. It is therefore beneficial to perform decomposition in the sinogram domain before reconstruction,^6^ although this requires a higher degree of spatial and temporal fidelity of the input data.^7^ Decomposition can also be integrated into the reconstruction to further improve performance,^8^ but this has not yet been implemented commercially due to significant increases in reconstruction time.

The concept of spectral CT was already described by Hounsfield in his seminal article from 1973.^9^ However, computational limitations and a lack of integrated solutions hindered clinical implementation. In the last decade, spectral CT has become more mainstream, with various commercial implementations.^10^

The first available spectral technique was a sequential DE CT scan. By making two sequential scans with different tube potentials, a DE dataset can be created without any technical adaptations to the scanner.^11^

The technique is associated with a temporal offset in the order of a single scan duration, which poses a problem in the presence of (involuntary) motion and dynamic contrast enhancement and limits the possibility to perform spectral decomposition on sinogram data.^6^ Also, integration with more complex acquisition protocols such as 4D CT is possible,^12^ but it remains challenging to minimize motion artifacts.

A particular type of sequential DE CT is split‐beam DE CT.^13^ In a helical scan with a pitch under 0.5, a two‐material filter allows for separation of the tube output into two separate energy levels. This technique allows for reduction of the temporal offset in comparison to sequential DE CT, but at the cost of spectral separation.^14^

Because of the limited temporal resolution and image quality, the use of sequential DE CT remained limited to certain niche applications,^15^ such as urinary calculi,^16^ gout,^17^ and hepatic steatosis.^18^ For RT, the use of these techniques is mainly limited to proton stopping power ratio (SPR) calculations.^19^, ^20^ It was only with the introduction of modern CT scanners providing integrated dose‐neutral solutions with high image quality that spectral CT became an established technique in radiologic departments.^21^

The first integrated iteration introduced an extra X‐ray source and detector pair. This technique allows for two simultaneous acquisitions at different kilovoltage peak (kVp) levels,^22^ but the angular offset will introduce an effective temporal offset in sinogram space of 25% of the rotation time. This offset can cause motion uncertainties and will introduce a geometrical offset in helical mode, which makes sinogram‐based decompositions challenging.^7^ An additional limitation of a dual‐source solution is that both detectors are competing for angular space.^22^ This implies an intrinsic limitation to the spectral field‐of‐view (FOV), which is problematic for dose planning.^12^

Another implementation rapidly switches the tube potential during gantry rotation,^23^ resulting in two scans with different energy levels. The advantage of this technique is that the temporal and geometrical offset between the scans is minimal, allowing sinogram‐based decomposition.^7^ However, this design poses a constraint on dose modulation, rotation speed, and scan speed to counteract for low energy flux which compromises the advantages over conventional CT.^24^

The most recent commercial iteration separates the signal by means of a dual‐layer detector.^25^ The top layer of the detector attenuates and detects the low‐energy photons, while the bottom layer detects the remaining high‐energy photons. A detector‐based solution avoids acquisition constraints and creates two energy datasets without any offset. It has a reduced spectral separation in comparison to other solutions,^26^ but with a spectral signal‐to‐noise ratio (SNR) that was at least comparable to other commercial solutions.^27^ A unique feature of this technique is that by recombining the signal from the two layers, a conventional CT scan can be created from the spectral data at the energy level of the acquisition (120 or 140 kVp), which facilitates direct comparison and adoption.

The term spectral CT and DE CT are often interchanged. In this article the term spectral CT has been chosen, since it is more general than DE‐CT and could also refer to other solutions like photon‐counting (PC) CT. Photon counting CT detects individual photons and measures their energy.^28^ This has the potential to provide CT data without electronic noise, improved tissue contrast, and improved image resolution.

Spectral CT imaging is gaining relevancy in the radiological clinic, since it improves image quality due to increase of image contrast^27^ and a reduction of beam‐hardening,^6^ image noise^29^ and metal artifacts.^30^ Furthermore, it allows for quantification of the concentration of contrast agents such as iodine.^31^ This is especially relevant for oncological application, since intravascular iodine is often used as a contrast agent for visualizing hyper‐ and hypo‐perfusing tumors. By assessing spectral images such as low‐keV VMI, virtual non‐contrast (VNC), and iodine concentration images, iodine contrast is enhanced and quantifiable, which helps in the detection and assessment of tumors, lymph nodes, and metastasis.^32^

The increased use of spectral CT in oncological radiology has sparked an interest within the radiation oncology community.^33^ However, despite having a potential in improved visualization and characterization of cancer,^32^ most of the past works in radiation oncology focused on improving dose calculation for brachytherapy and proton therapy.

The main purposes of this review article are to increase the radiation oncology community's knowledge in latest usage of spectral CT and encourage novel clinical applications. This review addresses the threefold benefits of spectral CT for RT, summarizing current and potential applications of spectral CT in the RT setting: improved dose calculation through reliable radiation physics, treatment certainty through improved disease visibility, and personalized treatment through physiological quantification.

## 2 IMPROVED DOSE CALCULATION THROUGH RELIABLE RADIATION PHYSICS

Spectral CT measurements are more quantitative than measurements from conventional CT. Conventional CT reports the integral attenuation of a spectrum of radiation through the body, with results that are dependent on tube output and affected by beam hardening.

Spectral CT can provide a wide variety of well‐defined results such as VMI, Z_eff_ and ED maps.^10^ These results are in principle independent on scanner parameters and (in case of sinogram decomposition) not associated with beam hardening, although residual errors may persist.^6^

The implementation of spectral CT data as a replacement of the conventional planning CT for dose planning can improve accuracy for different RT modalities and potentially reduce the need for phantom calibration to convert Hounsfield units (HUs) into ED values.^34^

### 2.1 Dose calculation improvements

The use of HU images from conventional CT is adequate for the estimation of the tissue attenuation in external beam RT with megavoltage (MV) photons, because attenuation in water for both MV^35^ and kV beams^36^ is mainly attributed to CS through ED. The large dose penumbra associated with MV external beam RT makes additional uncertainties less influential.^37^ However, dose delivery techniques with a steeper dose gradient, such as brachytherapy^38^ and proton therapy,^39^ require more precise modeling for optimal therapy planning.

### 2.2 Monte carlo simulations for brachytherapy

One of the first spectral applications described for RT was to use spectral CT for Monte Carlo simulations for brachytherapy.^38^ For (low‐energy) brachytherapy, the attenuation contribution of the PE and to a lesser extent Rayleigh scatter is much more prominent than for MV external beam RT. This makes the dose gradient sharper, thus also raising the need for quantification of the effective atomic number (Z_eff_) for precise dose calculations.^40^

Moreover, Monte Carlo simulation is in general more sensitive to correct modeling of physical properties of tissues than classical dose calculation with analytical methods, requiring detailed knowledge of the atomic composition of the underlying tissue.^41^ For this reason, most Monte Carlo techniques rely on organ tissue segmentations, with assumptions on the chemical makeup of each tissue type.

A method for classification of tissue groups based on conventional HU value ranges has been described by Schneider,^42^ and this technique has since been regarded as the gold standard. However, studies have demonstrated that conventional CT segmentations are associated with large uncertainties, due to materials having similar conventional CT values, but different physical properties, leading to large dose calculation errors.^43^ Spectral CT could improve these segmentations (see the section on visibility), and one study demonstrated that this could reduce local dose errors from +9% down to ±4% in brachytherapy with ^103^Pd.^44^

However, segmentations based on HU values do not account for interpatient variations in tissue composition, especially in the case of local tumor pathology. Therefore, direct parametrizations of spectral features like ED and Z_eff_ to model elemental mass fractions have been proposed for particle therapy.^45^ However, Z_eff_ is not a reliable quantity to reproduce the attenuation for tissues at energies under 50 keV, which poses challenges for modelling low‐energy brachytherapy.^46^ Therefore, other authors have proposed other models, like a three‐material water‐lipid‐protein decomposition,^47^ or a naive model with virtual materials through a linear model^48^ or principle component analyses.^41^ The impact of the latter technique on dose distribution was tested for brachytherapy and proved to give higher accuracy than a conventional CT solution.^49^

### 2.3 Particle treatment planning

Particle therapy has a much steeper dose delivery curve than conventional external photon therapy, due to the associated Bragg peak.^39^ This allows for more precise targeting, but also raises the bar for SPR estimation. Proton SPR estimations on conventional CT are associated with range uncertainties of 3%–3.5%.^50^ For photon therapy such uncertainties in attenuation would constitute local dose variations under 1%,^37^ but for proton therapy variations in SPR could lead to much larger dosimetric uncertainties due to dose shifts. Some studies report variations up to 7.8% with an average uncertainty of 2.1%,^20^ although in theory differences could be up to 100% due to a local dose shift.

There are multiple studies that have described how spectral images can be used to calculate SPRs for proton^50^ and heavy‐ion treatment.^51^ Most techniques use various implementations of the Bethe equation to convert spectral values of each voxel into SPR,^41^, ^52^, ^53^, ^54^, ^55^ although stoichiometric calibration can also be applied.^56^ Recently, convolutional neural networks have also been proposed to create SPR maps from spectral CT data.^57^

Spectral CT can lower the root mean square errors (RMSE) of proton SPR from 3% to below 1% uncertainty^50^ for proton beam therapy, although some articles report clinical errors of 2.2%^58^–2.4%.^59^ The reported reasons for this discrepancy are noise and residual beam hardening artifacts, mainly related to the used image‐based spectral decomposition technique.^60^

### 2.4 Dose calculation in the presence of contrast agent

The use of iodine as a contrast agent is a crucial tool in the visualization of tumors on CT.^61^ Iodine has strong attenuation of low KV radiation and thereby increases the HU of perfused areas on CT. This helps with visualization of relevant structures, but it compromises the radiation dose calculation for photon therapy,^62^ brachytherapy,^63^ and particle therapy.^64^ The associated increase of HU generally leads to an overestimation of the attenuation and stopping power, which translates to an underestimation of the dose during photon therapy^62^ or a dose range overshoot for particle therapy.^64^

These effects are dependent on the tissue depth and the proximity of organs with strong perfusion, being more prominent in abdominal regions than in areas like the head‐and‐neck and pelvis. For photon therapy, the uncertainty on dose estimations remains under 1% for most areas, but it can be larger than 2% for abdominal treatment areas.^65^ For proton therapy, the effects are more substantial and can result in dose shifts up to 10 mm.^66^

To avoid the influence of iodine contrast on the dose calculations, an additional CT without contrast is typically added to the acquisition protocol for dose calculation. However, this increases imaging dose to the patient and introduces position uncertainty from patient motion between two scans. Therefore, this additional scan is sometimes omitted when dose effects are anticipated to be limited,^67^ or the local HU values in regions with high iodine uptake are manually replaced with values of the surrounding tissue.^68^

Spectral CT provides the possibility to create VNC CT scans, with HU similar to conventional CT scans without iodine.^69^ Some studies have investigated the use of such scans for the purpose of treatment planning, reporting RMSE deviations of attenuation and stopping powers below 1%, rendering these deviations negligible.^70^, ^71^, ^72^

**FIGURE 1:**
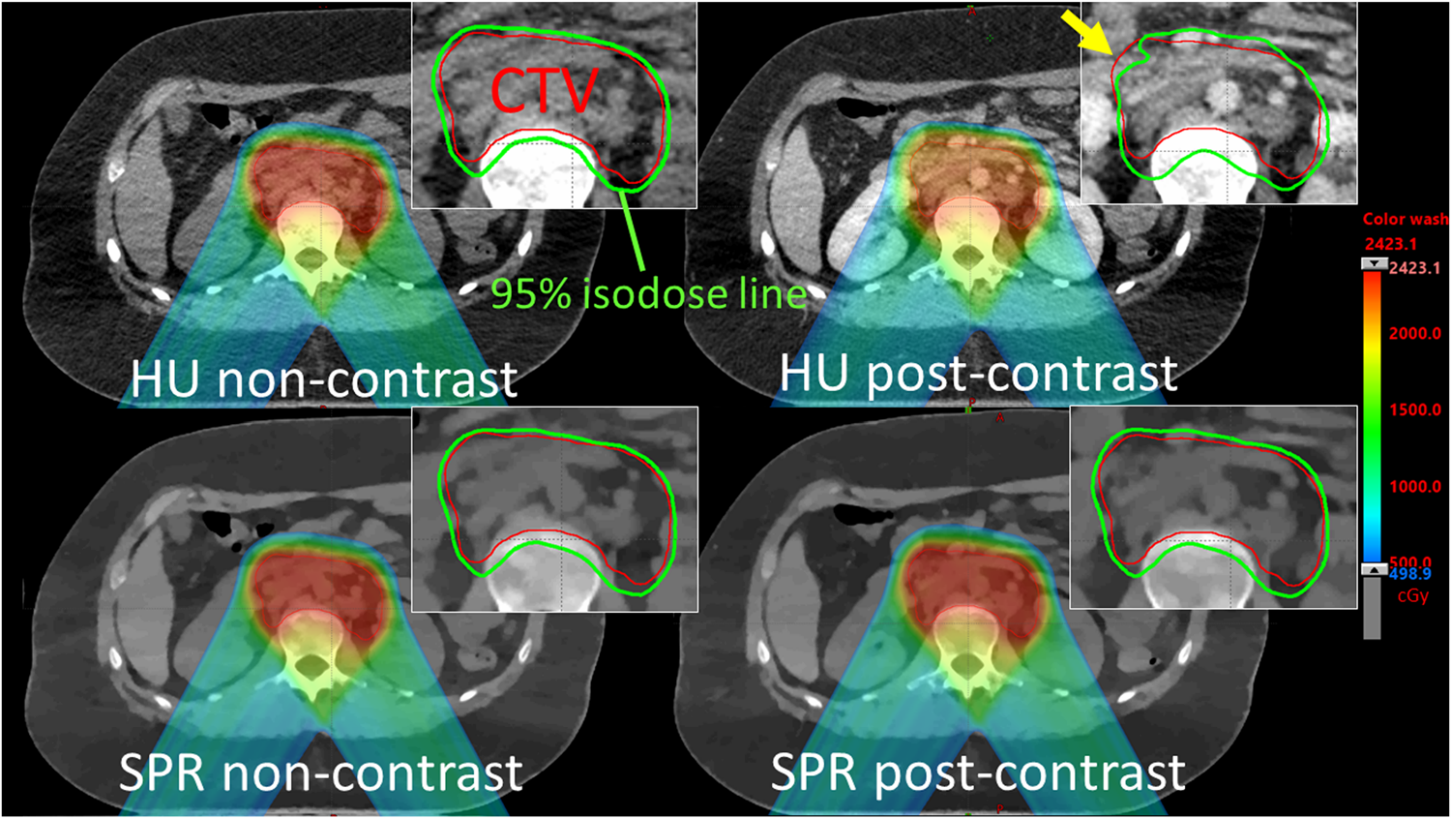
Spectral computed tomography (CT) can improve dose calculation in the presence of iodine contrast. This illustration shows comparisons of intensity‐modulated proton therapy plans designed on non‐contrast images and re‐calculated on post‐contrast images (Ates 2020). The isodose distributions are nearly identical when spectral stopping power ratio (SPR) images are used for dose calculations. However, the clinical target volume is underdosed when conventional post‐contrast conventional Hounsfield unit (HU) images as indicated by the arrow. (Images are courtesy of Dr. Ozgur Ates and Dr. Chiaho Hua from St. Jude Children's Research Hospital, Memphis, TN, USA)

For photon therapy, it is also possible to directly produce spectral ED maps for dose planning.^73^ Primarily, this would eliminate the need of calibration tables of HU to ED in photon therapy treatment planning. An additional advantage to this approach is that ED measurements are only marginally affected by iodine concentration. A clinically extremely high contrast concentrations of 20 mg/ml iodine is only associated with ED variations of about 5%,^34^ while organ perfusion will typically not exceed 2–5 mg/ml, leading to local uncertainties of 1% or less. Using the spectral ED map therefore eliminates significant planning uncertainty that is associated with iodine contrast in conventional CT planning.^74^ An illustration of this effect can be seen in Figure 1.

### 2.5 Metal artifact reduction

The presence of metal in the body can be a source of major artifacts in CT imaging due to photon starvation, beam‐hardening, and scatter effects.^75^ Foreign metal objects like hip prostheses,^76^ spinal fusion hardware,^77^ dental fillings^78^ as well as brachytherapy seeds^79^ can greatly compromise the calculation of local dose for different RT methods. These artifacts are often handled by manually overwriting affected regions with HU values of normal tissue.^80^

Most contemporary CT scanners provide metal artifact reduction (MAR) algorithms that can reduce these artifacts in conventional CT.^81^ MAR algorithms can be applied to conventional data by making assumptions on beam hardening and data that are missing due to photon starvation, and this can improve the accuracy of calculated dose.^82^

The use of spectral CT can also reduce metal artifacts.^76^ The reduction of metal artefacts is mainly attributed to the reduction of beam‐hardening artefacts in spectral CT.^83^ The overall artefact reduction is largest in high KeV VMI, since these have the strongest contribution of the high‐energy channel of the DE input data (resulting in minimized photon starvation). MAR algorithms can be combined with spectral high keV VMI to further optimize results.^84^

Reduction of metal artifacts through spectral CT can improve dose calculation for external photon^85^, ^86^, ^87^, ^88^ and brachytherapy.^89^ The impact in dose calculations effects varies largely between metal implant type and size. Although some reported a negligible dose advantage for photon therapy,^90^ others reported a reduction of average dose error from 4.4% for conventional CT to 1.1% for a spectral 180 keV.^91^

## 3 IMPROVED VISIBILITY LEADS TO INCREASED CERTAINTY

Conventional CT is still the dominant imaging modality in RT for target definition and disease staging,^92^ but other imaging modalities are quickly gaining relevancy. Conventional CT is associated with limited soft tissue contrast even in the presence of contrast agent and for this other imaging modalities with higher sensitivity and specificity, like positron emission tomography (PET)^93^ and magnetic resonance imaging (MRI),^94^ are becoming more important in RT.^95^

Spectral CT can improve the contrast of various neoplasms and provides insight on their malignancy.^32^ Spectral images generally yield reduced noise^96^ and beam hardening artifacts,^6^ and by selecting the right spectral results, the visibility of tumors can be optimized.

Low keV VMI maximizes the general iodine contrast in the scan,^27^ while high keV VMI data minimize metal artifacts.^30^ Z_eff_ can characterize materials based on their chemical composition,^97^ and an iodine map visualizes iodine concentrations specifically.^98^ These maps improve the visibility of primary tumors, metastases, and involved lymph nodes.

### 3.1 Tumor delineation

**FIGURE 2:**
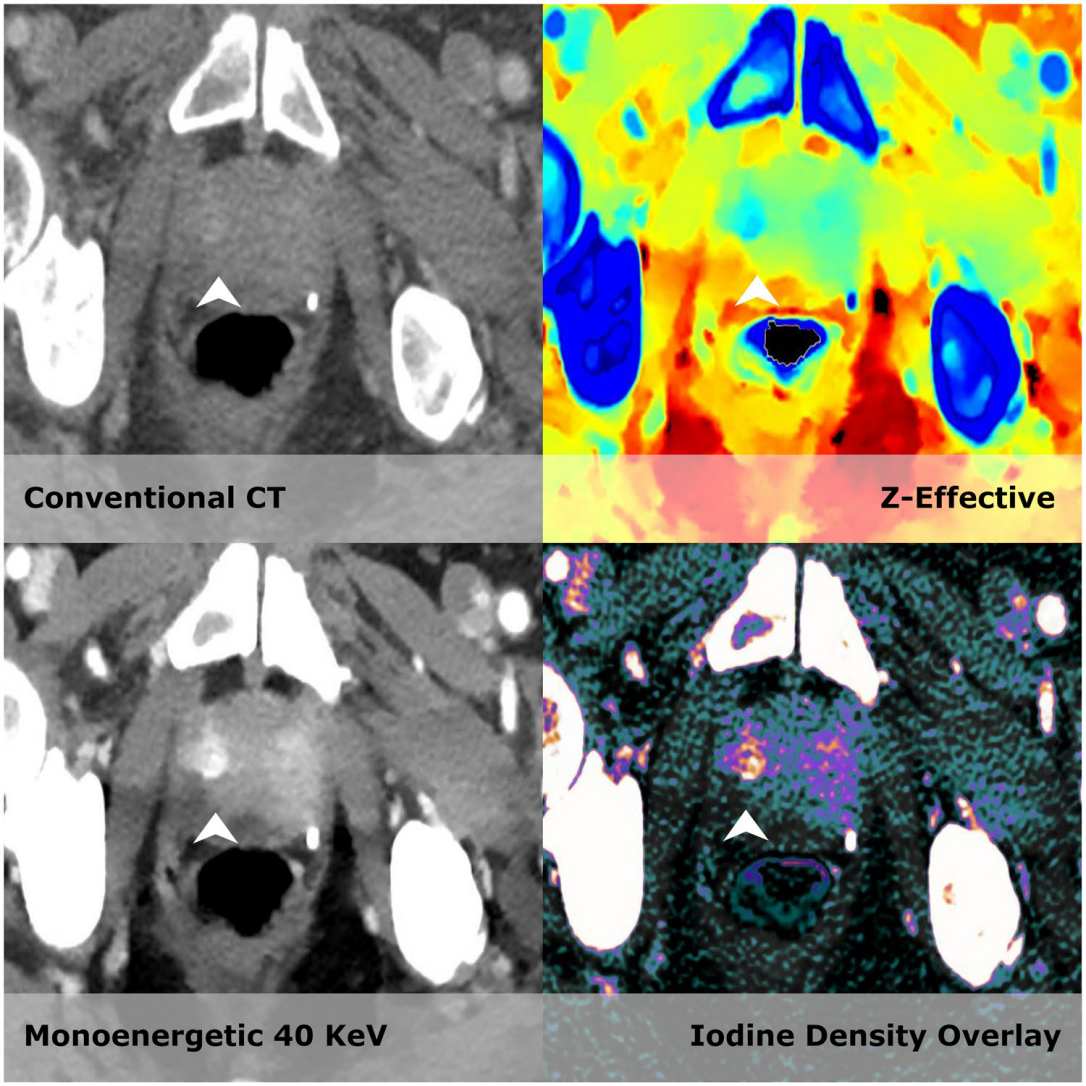
Computed tomography (CT)‐based radiotherapy (RT) treatment of the prostate often aims for the entire gland, because the tumor is usually hardly visible on conventional CT. Magnetic resonance imaging (MRI) data are therefore commonly added to define the exact tumor extent within the gland. This figure is an example of an 82‐year‐old male with biopsy‐proven prostate cancer (Gleason 3 + 4 = 7) in the right side of the gland (arrow), demonstrating that spectral CT allows for better discrimination of the tumor. It is very likely that this information can be used to improve treatment precision. (Images are courtesy of Dr. Michael Brun Andersen and prof. Dr. Finn Rasmussen from Aarhus University Hospital)

The use of iodine as an intravenous contrast agent can improve soft tissue contrast on CT. However, the increase of perfusion can often be very subtle (e.g., prostate tumors, see Figure 2, ^99^) or even hypodense in comparison to the surrounding parenchyma (e.g., pancreatic adenocarcinoma^100^). This lack of contrast can cause stark variations in target delineations of many treatment areas^101^ for head and neck,^102^ gastric,^103^ pancreatic,^104^ prostate,^105^ bladder,^106^ and even pulmonary^107^ cancers.

Various radiological studies have proven that spectral CT improves tumor visibility, by improving the tumor contrast for malignancies such as lymphomas,^108^ head and neck (H&N) lesions,^109^, ^110^, ^111^, ^112^ lung cancer,^113^ breast,^114^ pancreatic cancer,^115^, ^116^, ^117^ prostate cancer,^118^ gynaecological cancer,^119^ urothelial carcinoma,^120^ and hepatocellular carcinoma (HCC).^121^ All studies indicate that low keV VMI images yield increased contrast‐to‐noise (CNR) of the tumor in comparison to conventional CT. The increase of CNR was very dependent of tumor type as well as acquisition and reconstruction methods, ranging from 36.6% for H&N cancer^109^ to 62.3% for bladder cancer.^120^ Tumors located near metal implants are an exception, since high VMI images yield minimalization of metal artifacts.^122^, ^123^

Two articles compared tumor PET uptake to iodine contrasts of the tumor on spectral CT. One study compared the ability of spectral CT to predict microscopic invasiveness for non‐small cell lung cancers (NSCLCs) to that of ^18^F‐Fluorodeoxyglucose (FDG) PET, finding that the diagnostic performance of both modalities was similar.^124^ Another study found high correlation (*R* ^2^=0.82) between PET standard uptake values (SUVs) and multiple spectral CT features in patients with pancreatic adenocarcinoma, concluding that spectral CT is a potential surrogate for FDG in assessment of these tumors.^125^

Although a lot of articles claim to have investigated the impact of spectral CT on tumor delineation, most have merely investigated features like SNR and CNR. Only one study really reported the effect of image quality improvement on delineation variability. A comparison of spectral CT with MRI finds that 60 keV VMI provided a higher interobserver agreement for non‐skull base tumors in comparison to T1 with contrast and T2 weighted MRI.^126^ A limitation to this study is that the results are not compared to conventional CT, making it impossible to attribute the specific benefits of spectral CT over conventional CT.

### 3.2 Staging of lymph nodes and metastasis

Improved contrast not only increases the visibility of the main tumor, also metastasis and lymph nodes become more apparent, which is crucial for disease staging. Although staging is not typically performed in RT departments, it is still helpful to detect and assess the malignancy of lymph nodes and metastasis at the time of RT planning.

For the detection of metastasis, many of the relevant techniques are similar to the detection and delineation of the main tumor. However, for correct assessment of hyperdense findings, it is essential to discriminate iodine from other contrasts to determine malignancy.^32^ Spectral CT can be very useful for the detection and assessment of lung,^127^ liver,^128^ bone,^129^ brain,^130^ and adrenal metastasis,^131^ as well as local invasion of gastric^132^ and lung cancer.^133^

This is also the case for determination of lymph node involvement. For assessment of the lymph node stations, assessment of node size is not enough; it is crucial to assess texture, local perfusion, and the pattern of perfusion within the nodes.^134^, ^135^, ^136^ By visualization and quantification of iodine,^137^ spectral images help in the assessment the malignancy of lymph nodes in the abdomen,^138^ HCC,^139^ H&N,^140^, ^141^, ^142^ gastric^143^ ^,59^, pulmonary,^168^ colorectal,^144^ and rectal cancer.^145^, ^146^

Within RT, PET, and MRI currently play an integral role in RT staging of many cancer types. To our knowledge, there is no literature on direct comparisons between the performance of these modalities to spectral CT on staging. There are, however, two studies that demonstrate the additional value of spectral CT to PET in staging of single osteolytic metastases of resectable NSCLC^147^ and to both MRI and PET for staging of H&N cancer.^148^

### 3.3 Organs‐at‐risk delineation

Delineation of OAR is crucial for sparing of these organs, but especially in areas with complex anatomy like the head and neck, discrimination of different organs‐at‐risk can be challenging on conventional CT.^149^ But even in simpler anatomies, improved organ discrimination might help with automation of the delineation process.^150^

Spectral CT increases HU stability,^151^ and the concept of spectral fingerprinting^152^ might raise the possibility to describe tissue types, based on specific CT features, which would aid tissue segmentation for the purpose of OAR definition. A number of studies report that spectral CT improves the performance of artificial intelligence (AI) for the purpose of auto‐delineation for various abdominal organs,^153^ bones^154^ as well as OARs in the head.^155^, ^156^

## 4 QUANTIFICATION LEADS TO PATIENTS‐SPECIFIC CARE

CT is by nature a scanning technique that provides anatomical information. The introduction of a contrast agent to a conventional CT scan can add quantitative information on physiologic features like perfusion and ventilation. But in order to quantify local concentrations, a dynamic scan is necessary, complicating the acquisition protocol at an elevated dose.

Spectral material decomposition allows for quantification of local contrast agent concentrations in a single static scan, which potentially could be used as a replacement for dynamic scans for tumor differentiation, treatment response analyses, adaptive treatments, and elective strategies to save functional parts of organs‐at‐risk.

### 4.1 Tumor differentiation

Traditionally, RT aims to administer a homogeneous dose to the delineated tumor. However, it is known that hypoxia, proliferation, and other functional factors have a local influence on radiation sensitivity of cancers.^157^ Therefore, inhomogeneous dose can be prescribed by either giving a boost within a delineated tumor^91^, ^158^ or by abandoning delineation and use a concept of tumor probability^159^ or dose‐painting‐by‐numbers^160^ for local dose prescription based on functional imaging.

PET and MRI are primarily used for assessing local tumor physiology. The role of conventional CT has been limited in functional tumor differentiation, because static CT provides little functional information. There have been attempts to use dynamic contrast enhanced (DCE) CT^161^ for various RT applications, but due to increased imaging dose, limited scan FOV and general scan complexity associated with DCE CT, DCE MRI is generally favored instead.^162^ The use of local perfusion parameters from DCE CT or MRI has been described extensively for target definition in diseases like prostate cancer^163^ and pulmonary cancers.^164^

**FIGURE 3:**
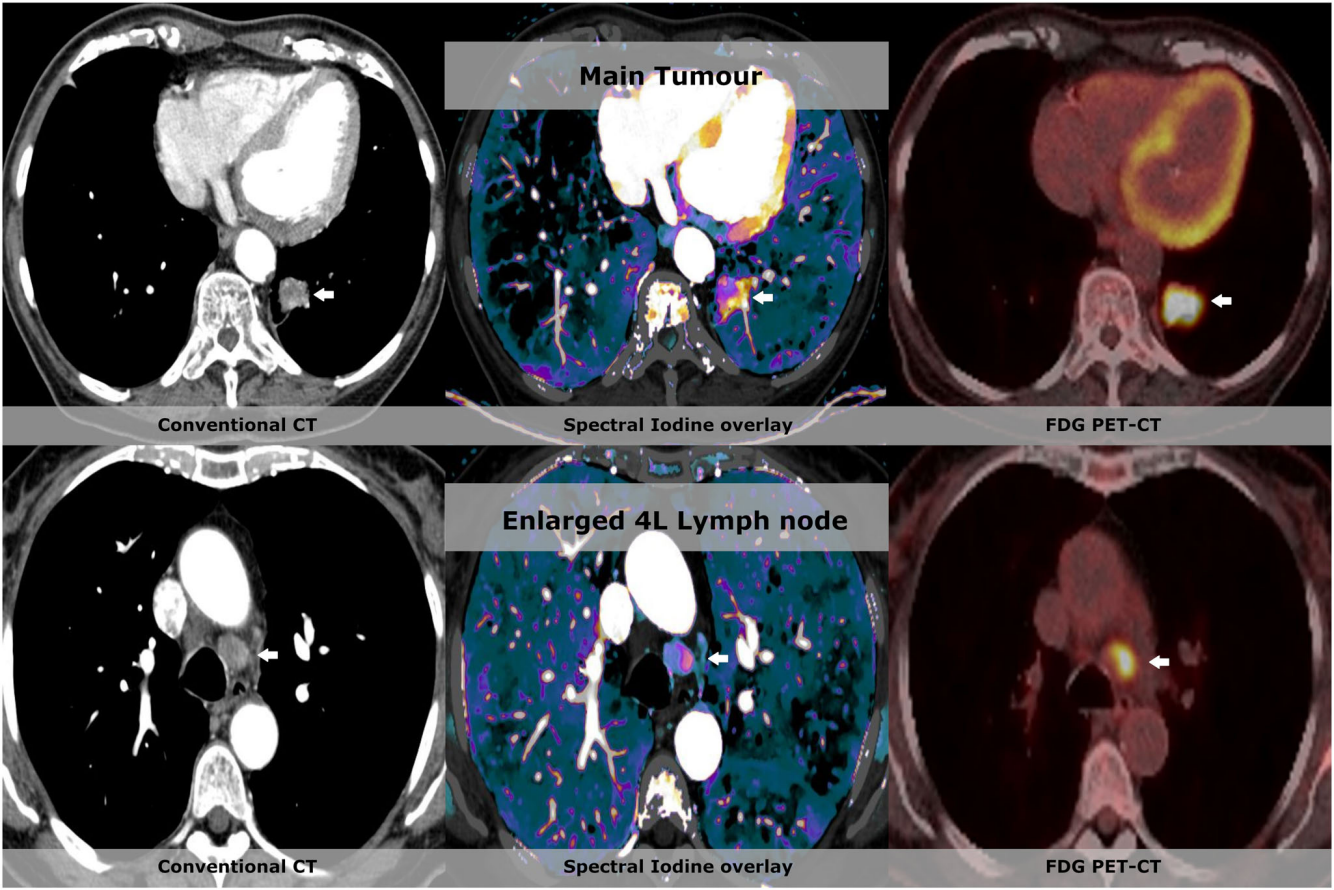
Spectral iodine quantification can be used to differentiate within the tumor and lymph nodes. This figure shows data from a 75‐year‐old male with lung cancer. In the top row, the conventional series show a 25 mm tumor paravertebral in the left lower lobe with slight enhancement. The iodine map shows that there is iodine uptake in the entire tumor with strong increased perfusion in the lateral and posterior part of the tumor, where corresponding positron emission tomography (PET) images show 8F‐Fluorodeoxyglucose (FDG) uptake in the entire tumor. The patient also had an enlarged 4L lymph node of 14 mm in short axis. The corresponding iodine overlay shows a similar perfusion and FDG uptake pattern in the left side of the lymph node. (Images are courtesy of Dr. Michael Brun Andersen and prof. Dr. Finn Rasmussen from Aarhus University Hospital)

Spectral CT allows quantification of local tumor perfusion from a static contrast enhanced CT, by quantification of the iodine concentrations at one timepoint (see Figure 3). Although this technique does not provide elaborate DCE features like vascular permeability and mean transit time, it is regarded as an indicator of local perfusion.^165^

For lung tumors, spectral iodine quantification values have been demonstrated to be associated with tumor differentiation^166^ and proliferation.^167^, ^168^, ^169^ Another study describes the use of normalized iodine concentration as a biomarker for the aggressiveness of rectal cancer.^170^ Potentially this information could be used for dose description, although to our knowledge, there is no study that describes this application.

It is, however, important to realize that timing of the scan is crucial. There are several studies that assess the correlation of FDG PET uptake to spectral CT iodine maps in lung cancer. One study found a positive correlation between the maximum SUV (SUV_max_) and the maximum iodine concentration (Iodine_max_) in late arterial phase,^171^ while another study finds a negative relationship between SUV_max_ and iodine concentrations in the venous phase.^172^

In a recent study, it was demonstrated that also distribution patterns can provide prognostic value for recurrence after SBRT of lung cancer.^173^ It was reported that the ratio between the low‐density area and the total tumor volume was negatively predictive for outcome, illustrating that heterogeneity of perfusion in the tumor might be relevant to the treatment plan.

### 4.2 Treatment response monitoring

Changes in tumor and normal tissue perfusion, during and after therapy, can be an indicator of treatment efficacy as well as damage inflicted to the surrounding parenchyma. For this reason, DCE MRI and CT are often used to monitor treatment effects of various treatment modalities on different cancer types.^174^, ^175^, ^176^

There have been a number of articles that describe that perfusion parameters can be used for treatment response of RT, specifically for various tumor types, like rectal,^177^, ^178^, ^179^ pulmonary,^180^, ^181^ cervical,^182^, ^183^, ^184^ H&N,^185^ and pancreatic cancers.^186^

Since spectral iodine maps are an indicator of local perfusion, they have been used to monitor targeted therapies for diseases like gastrointestinal stromal tumors^187^, ^188^ and NSCLC.^189^ More recently studies have demonstrated the feasibility of monitoring RT outcomes with spectral CT for lung,^190^ cervical,^191^ pancreatic,^192^ and H&N cancers.^193^, ^194^

In two studies, iodine quantification through spectral CT to assess treatment response of lung cancers to chemoradiotherapy and RT was compared to FDG PET. It was found that both modalities correlated well, indicating the feasibility for substitution of the PET follow‐up scan with a spectral CT exam.^195^, ^196^

Also, for rectal cancers spectral iodine quantification can be used for response evaluation. In a study from 2020, Sauter at al.^197^ demonstrated a very strong correlation (*r* = 0.73; *p* = 0.01) between the changes of both spectral iodine quantification and MRI‐based mean apparent diffusion coefficient (ADC), in follow‐up scans of patients that underwent radio‐chemotherapy. Since ADC MRI is considered the gold standard for response evaluation in rectal cancer and because of the strong correlation, they concluded that spectral iodine quantification could be a good alternative.

Another study investigated the potential use of spectral iodine maps for follow‐up imaging of NSCLC after chemoradiotherapy.^198^ In this study, a correlation was found between local iodine values in the tumor and disease progression as defined by RECIST criteria. Patients with disease progression also demonstrated higher iodine hotspots directly after treatment, indicating remaining vital tumor tissue.

### 4.3 Functional sparing of organs‐at‐risk

Functional discrimination within OARs might raise the possibility to spare healthy parts of OARs, over less‐functioning parts. Some studies have used ventilation^199^ and perfusion^79^ parameters to selectively spare healthy lung tissues.

The imaging standard for assessment of pulmonary function in diseases like chronic obstructive pulmonary disease is combined ventilation/perfusion single‐photon emission CT (SPECT). This technique, however, lacks high spatial resolution^200^ and general availability in RT clinics. For this reason, assessment of perfusion through other modalities, like PET and CT, is favorable, and it has been demonstrated that spectral iodine maps can be used as a replacement of perfusion SPECT, for the sparing of functional lung tissue.^201^

Some groups have also attempted to assess lung ventilation through conventional CT techniques like 4D CT^202^ and dynamic imaging with krypton or xenon gas as a contrast agent.^203^ Xenon quantification can be performed by conventional subtraction CT,^204^ but visualization and quantification are generally improved by spectral CT,^205^ even for dynamic scans^206^). By combining these data with spectral iodine perfusion scans, a ventilation/perfusion CT can be constructed.^207^, ^208^

Since iodine and xenon have similar attenuating features, it is not possible to discriminate them on a single spectral CT. However, by replacing iodine with gadolinium as an intravenous contrast agent, it has been demonstrated that it is feasible to acquire a ventilation/perfusion CT with one spectral scan through three‐material differentiation.^209^

The potential of sparing functional tissue is not limited to pulmonary treatments only. In a recent study, spectral iodine maps were used to minimize the dose to the functional parts of the liver.^210^ It was demonstrated that this was possible, without compromising target coverage.

## 5 DISCUSSION

Spectral CT is becoming an established technique in radiology. There is extensive and convincing proof that spectral CT adds relevant radiological information for various tumors.^32^, ^211^, ^212^, ^213^

The main application of spectral CT in RT is for the creation of SPR to improve dose calculation in proton therapy. However, by improving tumor contrast and adding functional information, spectral CT could potentially pose an alternative for PET and MRI, hereby reducing treatment cost and complexity. But although it is tempting to extrapolate this radiological value toward the RT clinic, there is a need to test this hypothesis in clinical studies in an RT setting.^211^

Although PC CT systems are not yet clinically available, and RT applications have not been tested, the values that are described in this article will likely apply to this technique as well. Especially with regard to tumor quantification and visualization, PC CT has large potentials, due to the ability to potentially use new contrast agents and better quantify tissue composition.^28^ For dose calculation, the improved quantification of tissue make‐up could improve for instance Monte Carlo simulation.

Recent usability improvements of spectral CT have facilitated clinical adoption,^214^ but it still needs to establish its position in radiation oncology.^33^ It has been critical for radiologic adoption of spectral CT that the technique does not negatively impact workflow and patient throughput.^214^

Sequential DE‐CT protocols are available on most contemporary CT simulators and are being used in RT for applications like SPR calculations,^19^ contrast enhancement,^156^ and 4D CT imaging.^12^ They might remain an alternative to the much more expensive integrated spectral solutions, in spite of their limitations in applications, quantitative features, and data integration.

An alternative approach would be to register an extra diagnostic spectral CT scan to the conventional planning CT, but this would complicate the workflow and render the conventional planning CT largely redundant. The use of a spectral CT scanner from a radiology department as a simulator could pose as a solution, but this could result in patient/staff scheduling complexity, since it would require interdepartmental cooperation. The scanners could furthermore compromise on specific RT needs, like bore size.

The optimal implementation option seems to be an integrated spectral solution, which is lacking in the current generation of CT scanners dedicated for RT simulation. An integrated spectral CT simulator would require significant design modifications, which can only be justified by considerable benefits for the majority of RT use cases. Spectral CT is currently mainly used in institutions that offer particle therapy for the promise of improved treatment planning, but it needs to demonstrate a broader benefit to exert a stronger impact on the radiation oncology community.

The increased disease visibility and quantifiability that are associated with spectral CT are potentially beneficial for all treatment modalities and might even be crucial for the future of CT simulation. However, most of these potentials require iodine as a contrast agent, which is problematic for sequential DE‐CT due to limitations in temporal resolution. The lack of an integrated spectral RT solution is therefore part of the reason that the application of spectral CT in RT is limited to applications that do not require high temporal resolution, like dose planning for particle therapy.^50^

Over the past two decades, the need for functional imaging in RT has increased both for staging,^215^ treatment planning,^95^ and response assessment.^92^ As a result, the demand for^93^, ^216^ and access to^217^ both PET and MRI in RT departments has increased significantly over the last two decades, thereby potentially reducing the role for CT to mere dose calculation and cone‐beam position verification.^218^ The integration of MR scanners during RT treatment,^219^ as well as the development of MR‐based synthetic CT images^220^, ^221^ enable an MR‐only workflow,^222^ which could potentially erode the value of CT in RT.^223^

Although both PET and MRI provide useful information, both bring limitations to a nimble RT clinic. Both modalities are expensive, complex, time‐consuming and require specialized staff that needs to be trained in the RT department or lend from other department to safely operate the scanners.^206^, ^224^, ^225^ MRI is associated with reduced geometric fidelity, and PET is associated with low intrinsic resolution. PET also requires expensive, short‐lived radioactive tracers and requires long preparation procedures of fasting and resting prior to the scan, which hamper integration into adaptive RT schemes.^226^ Furthermore, both PET and MRI have typical acquisition times in the order of 15–60 min, which limit throughput and pose a challenge for dynamic organs. In comparison to that, CT is much faster with acquisitions in the order of singular minutes.

Another issue for both MRI and PET is reproducibility. To be able to use quantitative results for local dose prescription, reproducibility of results between patients and vendors is paramount. Both PET^227^ and MRI^95^, ^216^ have a vast number of acquisition parameters that can influence measurements. There are many efforts to homogenize outcomes, but it remains challenging to assure reproducibility between patients and machines. This is problematic when local values are directly linked to local dose definition.^160^

CT results are much more reproducible, because the interaction of radiation with tissues is well defined.^36^ Spectral CT adds precision to conventional CT since it largely eliminates the effects of tube output and beam hardening on the results (in the case of sinogram‐based decomposition).^10^ This makes the results more reproducible and very suitable for direct dose prescription, although small differences between implementations of vendors exist.^228^

Spectral CT constitutes a wide variety of results, but how to practically implement these results into the RT clinic remains to be investigated. Because of the quantitative values of spectral CT results, spectral SPR or ED results could theoretically be sent straight to the treatment planning system to replace the planning CT, eliminating the need for a lookup table. But to incorporate different keV images, iodine and Z‐effective maps into the delineation process would require careful considerations to maximize efficiency and avoid incorrect use of images that jeopardize patient safety. It is likely that we could learn much from how different parametric images of PET and MRI results were introduced into treatment planning.

The development of AI could aid implementation of spectral CT. The performance of AI benefits from an increased number of parameters,^229^, ^230^ and the use of spectral CT will therefore likely improve the outcome of such algorithms. AI could help to manage large amounts of data, by aiding the radiation oncologist in their analyses. AI has already been applied to spectral CT data to detect lesions,^231^ predefine OARs,^155^ and assess involved lymph nodes.^232^ It is therefore likely that spectral CT and AI will develop in a symbiotic manner, where spectral CT improves the performance of different tasks performed by AI, while AI could make the associated data‐load manageable.

## CONFLICT OF INTEREST

The author is an employee of Philips Healthcare.

## Data Availability

Data sharing is not applicable to this article as no datasets were generated or analyzed during the current study.
